# Propulsion Calculated by Force and Displacement of Center of Mass in Treadmill Cross-Country Skiing

**DOI:** 10.3390/s22072777

**Published:** 2022-04-05

**Authors:** Shuang Zhao, Olli Ohtonen, Keijo Ruotsalainen, Lauri Kettunen, Stefan Lindinger, Caroline Göpfert, Vesa Linnamo

**Affiliations:** 1 Faculty of Sport and Health Sciences, University of Jyväskylä, 40014 Jyväskylä, Finland; zhaoshuangzs@hotmail.com (S.Z.); olli.ohtonen@jyu.fi (O.O.); keijo.s.ruotsalainen@gmail.com (K.R.); 2 Faculty of Information Technology, University of Jyväskylä, 40014 Jyväskylä, Finland; lauri.y.o.kettunen@jyu.fi; 3 Center of Health and Performance (CHP), Department of Food and Nutrition and Sport Science, University of Gothenburg, 40530 Göteborg, Sweden; stefan.lindinger@gu.se; 4 Department of Sport Science and Kinesiology, University of Salzburg, 5400 Salzburg, Austria; caro.goepfert@gmx.de

**Keywords:** propulsive force, V2-skating skiing technique, double-poling skiing technique

## Abstract

This study evaluated two approaches for estimating the total propulsive force on a skier’s center of mass (COM) with double-poling (DP) and V2-skating (V2) skiing techniques. We also assessed the accuracy and the stability of each approach by changing the speed and the incline of the treadmill. A total of 10 cross-country skiers participated in this study. Force measurement bindings, pole force sensors, and an eight-camera Vicon system were used for data collection. The coefficient of multiple correlation (CMC) was calculated to evaluate the similarity between the force curves. Mean absolute force differences between the estimated values and the reference value were computed to evaluate the accuracy of each approach. In both DP and V2 techniques, the force–time curves of the forward component of the translational force were similar to the reference value (CMC: 0.832–0.936). The similarity between the force and time curves of the forward component of the ground reaction force (GRF) and the reference value was, however, greater (CMC: 0.879–0.955). Both approaches can estimate the trend of the force–time curve of the propulsive force properly. An approach by calculating the forward component of GRF is a more appropriate method due to a better accuracy.

## 1. Introduction

Forces acting on a skier’s center of mass (COM) in a forward direction are propulsive forces, which are the primary mechanical determinants of an cross-country (XC) skier’s performance [1]. The position of skier’s COM can be obtained by using the marker-based motion capture system with a segmental method [2]. Thus, forces acting on a skier’s COM can be obtained by multiplying COM acceleration with the total mass of the skier, and this will indicate how athletes overcome resistive forces. However, the contribution of single pole and leg thrusts could not be revealed. Therefore, it is essential to compute forces acting on the COM from the ground reaction forces (GRFs) generated from skis and poles, separately.

Except for estimating the propulsive force with the forward acceleration of COM and the total mass, other approaches have been developed. One approach is to estimate the propulsive force as the forward-directed horizontal component of the three-dimensional (3D) GRFs from both skis and poles that act on a skier (F_net_) [3]. The roller skis [3,4,5], skis [6,7], and poles [3,6,7] equipped with force sensors have been used to measure the forces generated from skis and poles. Combined with the pole angle, ski angle, ski-edging angle, and the incline of the track or the treadmill, the propulsive force from skis and poles can be specified [1,3,8]. Therefore, questions related to the propulsive force, including the contribution of skis and poles in different techniques [3,9,10], and the comparison of different techniques [11,12] have been addressed. Another approach, demonstrated by Göpfert et al. [6], is to estimate the propulsive force with the forward component of translational force (F_pro_). The translational force was modeled as the component of the 3D resultant GRFs that acts in the direction from the point of force application (PFA) to a skier’s COM [6], and calculated by projecting the GRFs to the line defined by the COM and PFA.

The propulsive forces obtained with the two mentioned approaches (F_net_ and F_pro_) have been compared to the propulsive force calculated with COM acceleration from a motion analysis system (F) in [6]. As using the segmental method has been shown to be suitable for estimating the position of the COM in sports [2], F was considered as the reference value. The results indicated that the force–time curves of F_net_ and F_pro_ all showed high similarity when compared to the force–time curves of F during the leg skating push-offs on snow. F_net_ overestimated F, and F_pro_ was found to be a more appropriate approach to estimate F during leg skating push-offs [6]. However, whether F_net_ and F_pro_ could be used to estimate F, and which one is more accurate in other techniques, are still unknow. As XC skiing is a sport whose competition and training are normally performed on varying track topography and speed, whether F_net_ and F_pro_ could work steadily when estimating F at different terrain with different speeds need further investigation.

Therefore, the first aim of the present study was to obtain the force–time curves of F_net_ and F_pro_ with different skiing techniques and evaluate which can estimate the force–time curves of F better. As the use and importance of double poling (DP) and V2 skating (V2) as main techniques in XC skiing have increased for the past few years [12,13,14], DP and V2 skating techniques will be performed in this study. The second aim is to investigate which approach is more accurate when estimating F. Another aim is to explore the stability of the approaches to calculate F_net_ and F_pro_ by changing the speed and incline of the treadmill. We hypothesized that the force–time curves of F_net_ and F_pro_ all give comparable shape with F in both techniques [6]. We also hypothesized that F_net_ would give a considerable overestimation, and F_pro_ would be more accurate than F_net_, when estimating F in both DP and V2 techniques [6]. We further hypothesized that the approaches to calculate the F_net_ and F_pro_ would not be affected by the speed and incline of the treadmill in both techniques.

## 2. Materials and Methods

### 2.1. Participants

A total of 10 experienced male skiers (age: 29.4 ± 7.9 years; height: 181.4 ± 5.7 cm; weight: 77.9 ± 8.9 kg) who were familiar with treadmill roller skiing volunteered to participate in this study. The experimental protocol and all methods used in this study were approved by the Ethics Committee of the University of Jyväskylä. All participants provided written informed consent before the measurement and were free to withdraw from the experiments at any point.

### 2.2. Protocol

The anthropometric parameters needed for motion analysis (e.g., bilateral leg length, knee width, ankle width, shoulder offset, elbow width, and hand thickness) were measured first, and passive reflective markers were attached to the participants and equipment. Once the preparations were made, participants completed a 10–15 min warm-up roller skiing on the treadmill. Next, calibration was performed with the skier in a standing position and the treadmill at a 0° incline. Participants then performed the DP technique at five speeds (13, 15, 17, 19, and 21 km/h) on a 2° incline. The comfortable pole length for the DP technique was 1.56 ± 0.06 m. After the trials with varying speeds at a 2° incline, the DP technique was performed at three inclines (3°, 4°, and 5°) with a speed of 10 km/h. There was a 1 min rest between each speed and incline. When participants finished performing the DP technique, the pole length was adjusted to a comfortable length for the V2 skating technique (which was 1.63 ± 0.03 m in this study). The participants were given a short rest period while adjusting the pole length. The participants then performed the V2 technique on the treadmill. The protocol for speed and incline change was the same as during the DP test.

### 2.3. Data Collection

An eight-video-camera motion capture system (Vicon, Oxford, UK) and NEXUS 2.8.1 software were used to collect and record the 3D trajectories of reflective markers at a sampling rate of 150 Hz. The global coordinate system (GCS) was defined using the right-hand rule when the incline of the treadmill was 0°. The *X*-axis of the GCS was defined as the direction from side to side across the treadmill. The *Y*-axis of the GCS was the longitudinal axis of the treadmill. The *Z*-axis of the GCS was perpendicular to the ground, pointing upward. The GCS was calibrated according to Vicon’s specifications. A total of 58 passive reflective markers were used in this current study: 43 passive reflective markers were attached to the participants’ bodies, and 15 markers were attached to the equipment, including both skis (3 each), both poles (3 each), and the treadmill (3). Anthropometric measurements and the placement of markers on the participants’ bodies were conducted according to the XC model [6] used in previous studies. Measurements were performed on a motorized treadmill with a belt surface of 2.7 m wide and 3.5 m long (Rodby Innovation AB, Vänge, Sweden). The same pair of roller skis were used for both techniques (Marwe, SKATING 620 XC, wheel no. 0), with a resistance friction coefficient of μ = 0.025 measured before the measurement (Appendix A).

**Figure 1:**
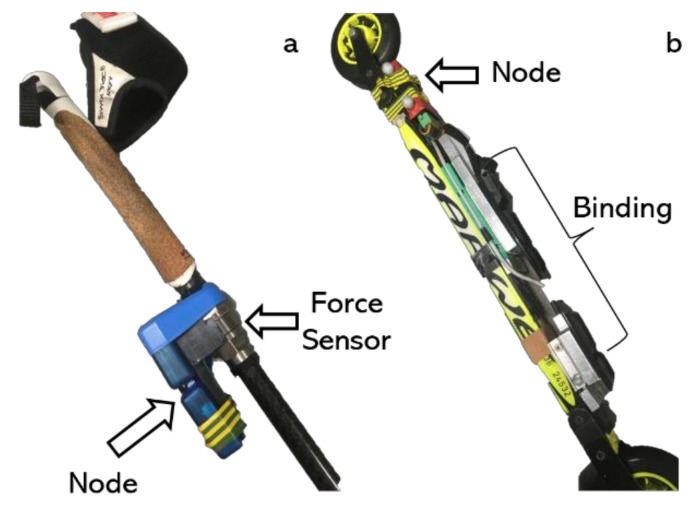
Equipment used in this study: (**a**) Pole force measurement sensor. (**b**) Force measurement binding.

Two custom-made pole force sensors (VTT MIKES, Technical Research Centre of Finland Ltd., Kajaani, Finland, Figure 1a) were used to measure the axial GRF from the poles. Two custom-made two-dimensional (2D) force measurement bindings (Neuromuscular Research Centre, University of Jyväskylä, Jyväskylä, Finland, Figure 1b) [15] were mounted on roller skis to measure the leg forces generated from roller skis. Both pole and leg forces were collected synchronously with the Coachtech online measurement and feedback system (Neuromuscular Research Centre, University of Jyväskylä, Jyväskylä, Finland) at a sample rate of 400 Hz. The force measurement bindings measured the vertical (F_skiz_) and mediolateral (F_skix_) forces and were calibrated before the measurement [15]. A trigger signal was sent from the Coachtech [16] to the motion capture system to mark the start of the force capture. The nodes for the pole force sensors and force measurement bindings were used to supply power and transmit data.

### 2.4. Data Reduction

**Figure 2:**
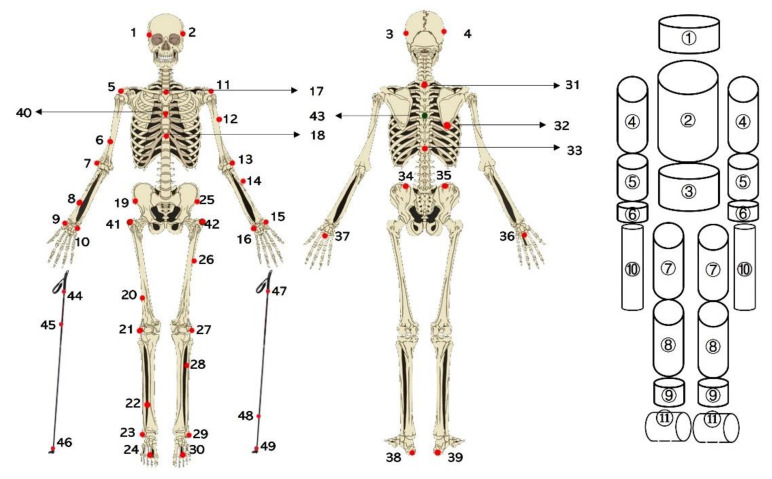
Marker placement on the subject and geometric model for segments in the XC model. The numbers 1–49 represent the placement of reflective markers on subjects and poles. The displacement of reflective markers on roller skis is shown in Figure 3. The numbers 1–39 are the markers used in the plug-in-gait (PIG) model. 1–43 are the markers used in the XC model [6] on one subject. The numbers 44–49 are the markers on the poles. ①–⑪ represent the head, thorax, abdomen and pelvis, upper arm, forearm, hand, thigh, shank, foot, pole, and roller ski, respectively.

Marker labeling and COM calculations were performed using NEXUS 2.8.1 software. The raw 3D trajectories of all reflective markers and the acceleration of COM were low-pass filtered (fourth-order, zero-lag, and Butterworth filter) with a cutoff frequency of 11.3 Hz [17]. The XC model [6], which contained the head, thorax, abdomen and pelvis, upper arms, hands, thighs, shanks, feet, skis, and poles, was used to calculate the whole-body COM. The marker placement on the subject and geometric model for the XC model is shown in Figure 2. The segmental anthropometric data were taken from Dempster’s study as described in Selbie et al. [18]. Force data were low-pass filtered (eighth-order, zero-lag, and Butterworth filter) with a cutoff frequency of 15 Hz [19]. Data filtering and parameter calculations were performed using MATLAB R2018a (MathWorks, Natick, MA, USA).

#### 2.4.1. Transforming the Forces Measured from the Force Sensor into the GCS and the PFA

**Figure 3:**
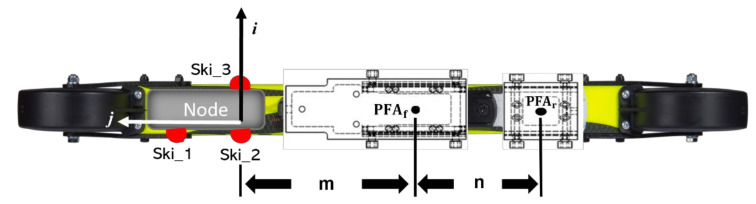
Displacement of markers, PFAs, and the definition of FCS ($\vec{i}$, $\vec{j}$, $\vec{k}$). Three markers (Ski_1, Ski_2, and Ski_3) were attached to the side of the node. The node for power supply and data transmission was attached to the front part of the roller ski. The surface defined by the markers was parallel to the roller ski surface. $\vec{i}$ was defined by Ski_3 and Ski_2. Another unit vector ($\vec{r}$) located on the surface of the roller ski was defined by Ski_1 and Ski_2. The surface norm, which was the $\vec{k}$ of FCS, was the cross product of $\vec{i}$ and $\vec{r}$. The last unit vector $\vec{j}$ was computed by using the right-hand rule with $\vec{k}$ and $\vec{i}$. The PFA_f_ and PFA_r_ were the points of force application of the front and rear sensors, respectively. The distance between Ski_2 and PFA_f_ was m, and the distance between PFA_f_ and PFA_r_ was n.

The forces generated from the roller ski force coordinate system (FCS) were transformed into the GCS. The unit vector of each axis of FCS ($\vec{i}$, $\vec{j}$, $\vec{k}$) was identified by markers on the roller ski (Figure 3). The transformation from the roller ski system to the GCS is given by

(1) $$\left[\begin{array}{c} F_{x}\\ F_{y}\\ F_{z} \end{array}\right]=R^{\prime }\left[\begin{array}{c} F_{\mathrm{skix}}\\ 0\\ F_{\mathrm{skiz}} \end{array}\right]$$

where F_x_, F_y_, and F_z_ are the components of forces generated from legs ($\vec{F_{r}}$) in the GCS. $R^{\prime }$ [18] is the rotation matrix from FCS to GCS.

The PFA is needed for calculating the translational force introduced by Göpfert et al. [6]. The displacement of the PFA along the binding (PFA_ski_) was calculated from the force distribution between the front and rear sensors of the binding [20] over time. The PFA for each part of the binding (PFA_f_ and PFA_r_) was defined as the center of each sensor. The distance between the marker Ski_2 and PFA_f_ (m), and the distance between PFA_f_ and PFA_r_ (n) were measured before the measurement. The displacements of PFA_f_ and PFA_r_ in GCS were obtained by moving the midpoint of Ski_2 and Ski_3 along the opposite direction of $\vec{j}$. The moving distances were m and m+n, respectively. The mediolateral sway of PFA_ski_ on ski binding was not considered in this study. Thus, the PFA_ski_ moved between the PFA_f_ and PFA_r_ (Figure 3).

The measured axial pole forces ($\vec{F_{p}}$) were considered the GRFs acting along the pole from the tip to the top of the pole and expressed that way in the GCS. The magnitude of $\vec{F_{p}}$ was collected using a pole force sensor. The direction of $\vec{F_{p}}$ was defined using the reflective markers that were attached to the pole. The PFA of poles (PFA_p_) was defined as the intersection of the plane of the treadmill and the long axis of the pole. The plane of the treadmill was defined using the three markers attached to the treadmill.

#### 2.4.2. The Reference Force, the Total Resultant Force, and the Translational Force

As using the segmental method has been shown to be suitable for estimating the position of the COM in sports [2], forces calculated by COM acceleration ($\vec{a}$) multiplied the total mass of the subject, and the equipment was the reference force ($\vec{F}$) in this study.

One approach to estimate forces acting on skier’s COM is to calculate the total resultant force ($\vec{F_{\mathrm{net}}}$) without considering the position of COM. $\vec{F_{\mathrm{net}}}$ is calculated as

(2) $$\begin{array}{c} \vec{F_{\mathrm{net}}}=\vec{F_{r}}+\vec{F_{p}}+\vec{F_{\mathrm{friction}}}+\vec{G} \end{array}$$

where $\vec{G}$ is the gravitational force of each participant and all the equipment. $\vec{F_{\mathrm{friction}}}$ is the frictional force between the roller ski and the treadmill, which was directed along the path of the ski motion, and the magnitude was computed by multiplying μ with $F_{\mathrm{skiz}}$.

**Figure 4:**
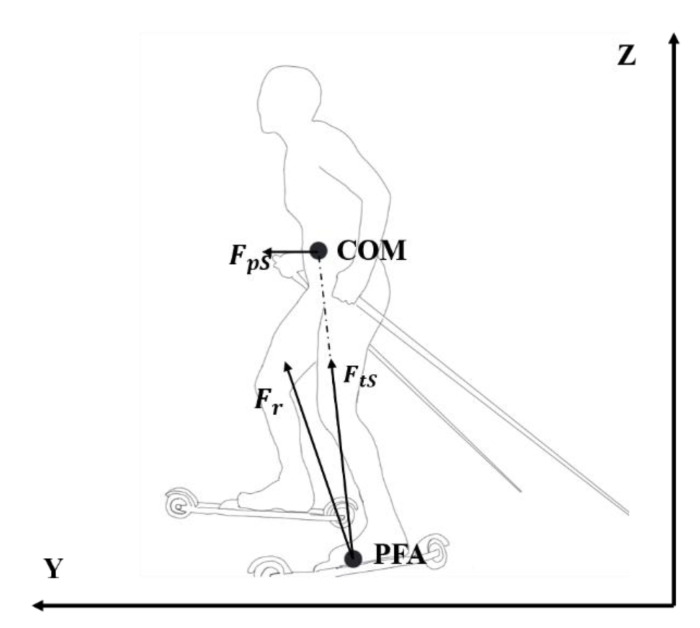
Diagram of force decomposition from skis. $F_{r}$ is the resultant force generated from legs. $F_{\mathrm{tS}}$ is the translational component, which went through the COM. $F_{\mathrm{pS}}$ represents the propulsion generated from legs in the forward direction.

Another approach to estimate forces acting on skier’s COM is to calculate the total translational force (the mechanical principle of translational force, see Appendix B). The translational force is the share of the resultant GRF acting in the direction from PFA to COM. The translational force from skis ($\vec{F_{\mathrm{tS}}}$, Figure 4) is the share of ski GRF ($\vec{F_{r}}$) acting in the direction defined from PFA_ski_ to COM and is calculated from

(3) $$\begin{array}{c} \vec{F_{\mathrm{tS}}}=\left(\vec{F_{r}}•\vec{u}\right)\vec{u} \end{array}$$

where $\vec{u}$ is the unit vector determined from PFA_ski_ to COM. The translational force from poles ($\vec{F_{\mathrm{tP}}}$) is calculated from

(4) $$\begin{array}{c} \vec{F_{\mathrm{tP}}}=\left(\vec{F_{p}}•\vec{v}\right)\vec{v} \end{array}$$

where $\vec{v}$ is the unit vector determined from PFA_p_ to COM. The total translational force ($\vec{F_{\mathrm{pro}}}$) is the sum of the translational force from the legs, poles, and the resistance. Thus, $\vec{F_{\mathrm{pro}}}$ can be computed as

(5) $$\begin{array}{c} \vec{F_{\mathrm{pro}}}=\vec{F_{\mathrm{tS}}}+\vec{F_{\mathrm{tP}}}+\vec{F_{\mathrm{friction}}}+\vec{G} \end{array}$$

As forces acting on skier’s center of mass (COM) in forward direction are the propulsive forces, the Y component of $\vec{F}$, $\vec{F_{\mathrm{net}}}$, and $\vec{F_{\mathrm{pro}}}$ (F, F_net_, and F_pro_) was compared and analyzed in the present study.

#### 2.4.3. Cycle Definition and Analyzed Parameters

**Figure 5:**
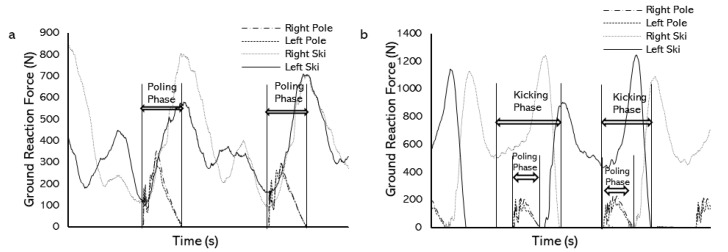
Definition of the force producing phase of DP and V2 techniques: (**a**) GRFs from skis and poles in the DP technique and the definition of the poling phase. (**b**) GRFs from skis and poles in the V2 technique and the definition of the kicking phase.

A total of 10 consecutive poling phases for each DP technique trial and 10 consecutive kicking phases (5 left ski kicking and 5 right ski kicking) for each V2 technique trial were analyzed. The poling phase was defined as the period from the start of the pole ground contact to the end of the pole ground contact (Figure 5a). The kicking phase was defined as the ski force minima until the end of ground contact [7] (Figure 5b). The forces of skis and poles from both the left and right sides were included while calculating the total propulsion in both techniques.

The positive square root of the adjusted coefficient of multiple determination, which is the adjusted coefficient of multiple correlation (CMC, 0 < CMC < 1) [21,22], was calculated for evaluating the similarity of force–time curves. One comparison was between F_net_ and F force–time curves. The similarity between F_net_ and F was represented by CMC_net_. The mean force difference and mean absolute force difference between F_net_ and F were $M_{F_{\mathrm{net}}-F}$ and $M_{\left|F_{\mathrm{net}}-F\right|}$. Another comparison was between F_pro_ and F force–time curves. The similarity between F_pro_ and F was represented by CMC_pro_. The mean force difference and mean absolute force difference between F_pro_ and F were $M_{F_{\mathrm{pro}}-F}$ and $M_{\left|F_{\mathrm{pro}}-F\right|}$. The mean force differences and mean absolute force differences were computed over force curves averaged over 10 force-producing phases. The mean force differences, which are $M_{F_{\mathrm{net}}-F}$ and $M_{F_{\mathrm{pro}}-F}$, were calculated to provide descriptive statistics only. The forces in this study were presented as values relative to body weight (%BW).

### 2.5. Statistical Analyses

A two-way mixed factorial ANOVA was performed. The dependent variables of the analyses were (1) CMCs and (2) mean absolute force differences. The independent variables were the speed (or the incline) of the treadmill and the comparisons (i.e., comparison between F_net_ and F and comparison between F_pro_ and F). The speed (or the incline) of the treadmill was treated as the within-subject factor, and the comparison pair was treated as the between-subject factor. The EMMEANS subcommand with the Bonferroni adjustment in SPSS was used to perform the pairwise comparisons of the dependent variable when interactions were detected [23], and the effect size (_p_η^2^) was calculated for further evaluation. The level of statistical significance was set at α = 0.05. All data are presented as mean ± standard deviation (SD). Data analyses were conducted using version 23.0 of the SPSS program package for statistical analysis (SPSS Inc., Chicago, IL, USA).

## 3. Results

**Figure 6:**
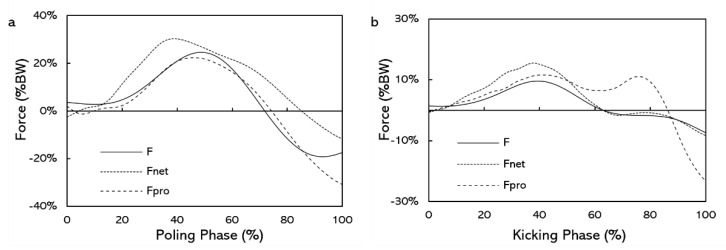
Force-time curves of F, F_net_, and F_pro_: (**a**) DP technique, (**b**) V2 technique. Values are averaged over 10 force-producing phases of one subject from each technique (speed of the treadmill was 19 km/h; incline of the treadmill was 2°).

The force–time curves of F, F_net_, and F_pro_ for the DP and the V2 techniques are shown in Figure 6. The interaction effect (comparison * speed) was significant on CMC with the DP technique (*p* = 0.038), but not with the V2 technique (*p* = 0.988). CMC_pro_ did not differ from CMC_net_ at any speed in the DP technique (*p* ≥ 0.106, Table 1). With the V2 technique, the overall CMC_pro_ was about 5% lower than CMC_net_ (*p* = 0.011, Table 1). The interaction effect (comparison * incline) was not significant on CMC in the DP technique (*p* = 0.620) but was significant in the V2 technique (*p* = 0.042). In the DP technique, the main effect of comparison on CMC was not significant (*p* = 0.218, Table 1). In the V2 technique, CMC_net_ was significantly greater than CMC_pro_ at 3° (*p* = 0.042, Table 1).

**Figure 7:**
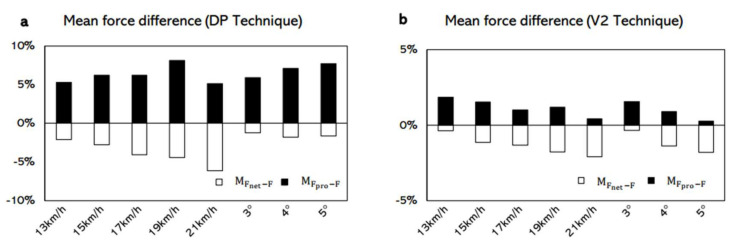
Mean force difference over force producing phases in DP and V2 techniques (%BW). $M_{F_{\mathrm{net}}-F}$ represents the difference between F and F_net_ and is calculated by F–F_net_. $M_{F_{\mathrm{net}}-F}$ lower than zero indicates that F_net_ is greater than F. $M_{F_{\mathrm{pro}}-F}$ represents the difference between F and F_pro_ and is calculated by F–F_pro_. $M_{F_{\mathrm{pro}}-F}$ greater than zero indicates that F_pro_ is lower than F.

On average, the $M_{F_{\mathrm{net}}-F}$ was lower than zero and the $M_{F_{\mathrm{pro}}-F}$ was greater than zero (Figure 7) for both the DP and V2 techniques at any speeds and any inclines. The interaction effect (comparison * speed) was significant on the absolute mean force difference with the DP technique (*p* = 0.025) but not with the V2 technique (*p* = 0.165). In the DP technique, $M_{\left|F_{\mathrm{net}}-F\right|}$ was 24% lower than $M_{\left|F_{\mathrm{pro}}-F\right|}$ at 15 km/h (*p* = 0.028, Table 2). For the V2 technique, the overall $M_{\left|F_{\mathrm{pro}}-F\right|}$ was about 37% greater than $M_{\left|F_{\mathrm{net}}-F\right|}$. The interaction effect (comparison * incline) was not significant on absolute mean force difference in the DP technique (*p* = 0.393) but was significant in the V2 technique (*p* = 0.016). In the DP technique, the overall $M_{\left|F_{\mathrm{net}}-F\right|}$ was about 39% lower than $M_{\left|F_{\mathrm{pro}}-F\right|}$. With the V2 technique, $M_{\left|F_{\mathrm{net}}-F\right|}$ was significantly lower than $M_{\left|F_{\mathrm{pro}}-F\right|}$ at 3° and 4° (*p* ≤ 0.013, Table 2).

With the DP technique, CMC_pro_ was independent from the speed (*p* = 0.371, Table 1). However, CMC_net_ decreased significantly at 21 km/h when compared to CMC_net_ at 13, 15, and 17 km/h (*p* ≤ 0.046). The overall CMC increased by about 2% from 3 to 5°. Both $M_{\left|F_{\mathrm{net}}-F\right|}$ and $M_{\left|F_{\mathrm{pro}}-F\right|}$ increased with the increasing speed of the treadmill (*p* < 0.001, *p* < 0.001, Table 2). The overall absolute mean difference increased by 23% from 3 to 5°. With the V2 technique, the overall CMC decreased by about 2% from 13 to 21 km/h. CMC_net_ was independent of the incline of the treadmill (*p* = 0.042, Table 1). CMC_pro_ increased from 3 to 5° (*p* = 0.007, Table 1). The overall absolute difference increased by 33% from 13 to 21 km/h. $M_{\left|F_{\mathrm{net}}-F\right|}$ was dependent on the incline of the treadmill (*p* = 0.014, Table 2), and $M_{\left|F_{\mathrm{pro}}-F\right|}$ was independent of the incline of the treadmill (*p* = 0.577, Table 2).

## 4. Discussion

The results of this study support our first hypothesis that the force-time curves of F_net_ and F_pro_ all give comparable shape with F in both techniques. In the DP technique, CMC_pro_ ranged from 0.907 to 0.936, CMC_net_ ranged from 0.883 to 0.955 and did not differ from CMC_pro_ (Table 1). In the V2 technique, CMC_pro_ ranged from 0.832 to 0.900, and CMC_net_ ranged from 0.879 to 0.922 (Table 1). The CMC depicting the similarity between waveforms and CMC close to 1 indicated that the curves involved were similar [21,22]. Therefore, the shapes of force-time curves of F_pro_ and F_net_ all showed similar to force-time curves of F, and both could be used to describe the shape of F during the poling phase of the DP technique and the kicking phase of the V2 technique. In addition, in the V2 technique, CMC_net_ was 5% higher than CMC_pro_ while changing the speed (Table 1), indicating that the force-time curves of F_net_ was more comparable to the force-time curves of F than F_pro_ while using the V2 technique. Consequently, F_net_ appears to be more appropriate for determining the trend of the forward acceleration in the V2 technique.

The results of this study partly support our second hypothesis that F_net_ would give a considerable overestimation and F_pro_ would be more accurate than F_net_ when estimating F in both the DP and V2 techniques. In this present study, the F_net_ had a considerable overestimation when estimating F in both the DP and V2 techniques, but F_pro_ was not more accurate than F_net_ in both techniques. The mean force differences over force curves between F_pro_ and F ($M_{F_{\mathrm{pro}}-F}$), as well as F_net_ and F ($M_{F_{\mathrm{net}}-F}$), were computed (Figure 7). The mean force differences in this study indicated that, on average, F_net_ would overestimate ($M_{F_{\mathrm{net}}-F}$ < 0, Figure 7) the F in both the DP and V2 techniques. F_net_ was calculated from the GRF directly. The costs associated with the transformations of energy [24] between each segment and the elastic potential energy of the muscle were not taken out from F_net_. Thus, a considerable difference in F_net_ and F may exist. The F_pro_ underestimate ($M_{F_{\mathrm{pro}}-F}$ > 0, Figure 7) the F, but it was not more accurate than F_net_ in both techniques. F_pro_ was calculated by combining the GRF and the position of COM. The resultant GRF was subdivided into a translational component, which acted through the COM, and a rotational component, which was always perpendicular to the translational component [6,25]. Because the rotational component will not have a translational effect on the COM, when F_pro_ was calculated, the rotational component was not involved. Therefore, the forward component of the translational component might underestimate the forward acceleration in both the DP and V2 techniques. The absolute mean force differences (Table 2) were computed to evaluate which force-time curve was closer to the reference one. A smaller absolute mean force difference indicates a force-time curve closer to the reference curve and further shows a relatively higher accuracy. The results of this study showed that with both the DP and V2 techniques, the absolute mean force difference between F_pro_ and F were greater than or have no difference with the absolute mean force difference between F_net_ and F. This indicates that the force-time curves of F_net_ were closer to or have no difference with the force–time curves of F. Thus, F_pro_ was not more accurate than F_net_.

The results of this study do not support our third hypothesis that the approaches to calculate the F_net_ and F_pro_ would not be affected by the speed and incline of the treadmill in both techniques. The approaches to calculate the F_net_ and F_pro_ were all influenced by the speed or the incline of the treadmill. As there was a balance of forces under laboratory conditions with no air resistance, constant friction coefficient, and constant gravitational force, the total external force remained constant when the speed was changed. The gravity component parallel to the treadmill surface increased with the incline [19]; thus, more forces were needed at a steeper incline. It is impossible to have an exact reproduction of the reference value F; however, if the methods for calculating F_net_ and F_pro_ were independent from the speed and the incline of the treadmill, the CMC_net_, CMC_pro_, and the absolute mean force difference over the force-generating cycle should remain constant in both techniques. The CMCs in this study were somehow affected by the speed and incline of the treadmill in both the DP and V2 techniques. In addition, the results of this study showed that the absolute mean force differences between F_net_ and F ($M_{\left|F_{\mathrm{net}}-F\right|}$) and between F_pro_ and F ($M_{\left|F_{\mathrm{pro}}-F\right|}$) were all affected by the speed of the treadmill regardless of whether the DP or V2 technique was performed (Table 2). The absolute mean force differences increased with increasing speed (Table 2), which means that although F_pro_ and F_net_ can be used to estimate the force-time curve of F, they do not remain stable when the speed changes. Thus, when investigating how F adapts to increasing speed by using F_net_ or F_pro_, the increasing mean force differences should be considered. Both $M_{\left|F_{\mathrm{net}}-F\right|}$ and $M_{\left|F_{\mathrm{pro}}-F\right|}$ increased when the DP technique was used while increasing the incline of the treadmill (Table 2). However, when the V2 technique was used, $M_{\left|F_{\mathrm{net}}-F\right|}$ was affected by the increasing incline, and the significant increase was only found at the steepest incline (Table 2), but $M_{\left|F_{\mathrm{pro}}-F\right|}$ was not influenced by the incline of the treadmill. Thus, compared to F_net_, F_pro_ was more stable when estimating F while changing the incline of the treadmill.

Therefore, when considering the whole poling phase in the DP technique, both F_pro_ and F_net_ are appropriate for estimating the trend of F. The similarity between the F_pro_ and F is stable while changing the speed in the DP technique. However, F_net_ has better accuracy than F_pro_ when the speed and the incline is changed. When considering the whole kicking phase in the V2 technique, the trend of F_net_ fits F better. However, the similarity between the F_pro_ and F is stable in the V2 technique when the incline is changed. As the result in the DP technique, F_net_ also has better accuracy than F_pro_ in the V2 technique. There are some limitations of this study. The calculation of the COM is dependent on the assumed mass distributions. Although this has been proved to be suitable for estimating the position of COM in sports, it can still cause the golden standard of the reference to be inaccurate. In addition, the PFAs of the leg force and pole force were estimated points, and this may also have some effects on the accuracy of F_pro_. Furthermore, the added measurement equipment could have affected skiing performance.

## 5. Conclusions

The present study evaluated two approaches for estimating the total propulsive force on skier’s COM. Both approaches can estimate the trend of the force-time curve of the propulsive force properly. Although both had a considerable overestimation; an approach by calculating the forward-directed horizontal component of 3D GRF is a more appropriate method due to a better accuracy. Future studies could investigate the contribution of skis and poles to forward COM acceleration by calculating the propulsive force from skis and poles separately. Moreover, as for the gliding phase that exists in XC skiing, the velocity at the end of the force generating phase is important. Future studies could also investigate the contributions of skis and poles to velocity change separately.

## Figures and Tables

**Table 1:** Mean and standard deviation of the CMC for the DP technique (*n* = 9) and the V2 technique (*n* = 10).

		DP Technique				V2 Technique			
		CMC_net_	CMC_pro_	*p*-Value	Pη^2^	CMC_net_	CMC_pro_	*p*-Value	Pη^2^
Speeds	13 km/h	0.935 ± 0.022	0.910 ± 0.038	0.106 ^b^	0.155	0.901 ± 0.048	0.853 ± 0.043		
	15 km/h	0.933 ± 0.023	0.916 ± 0.034	0.230 ^b^	0.089	0.908 ± 0.047	0.862 ± 0.050		
	17 km/h	0.920 ± 0.030	0.919 ± 0.030	0.951 ^b^	0.001	0.905 ± 0.040	0.861 ± 0.035	0.011 ^a^	0.309
	19 km/h	0.901 ± 0.045	0.908 ± 0.046	0.778 ^b^	0.005	0.885 ± 0.045	0.837 ± 0.047		
	21 km/h	0.883 ± 0.058 ^1,2,3^	0.907 ± 0.042	0.330 ^b^	0.059	0.879 ± 0.044	0.832 ± 0.041		
	*p*-value	0.043 ^d^	0.371 ^d^			0.008 ^c^			
	Pη^2^	0.509	0.264			0.216			
Inclines	3°	0.933 ± 0.024	0.914 ± 0.046			0.911 ± 0.032	0.856 ± 0.073	0.042 ^b^	0.210
	4°	0.946 ± 0.016	0.932 ± 0.033	0.218 ^a^	0.093	0.922 ± 0.041	0.896 ± 0.044 *	0.179 ^b^	0.098
	5°	0.955 ± 0.015	0.936 ± 0.037			0.912 ± 0.047	0.900 ± 0.055 *	0.617 ^b^	0.014
	*p*-value	0.001 ^e^				0.479 ^f^	0.007 ^f^		
	Pη^2^	0.464				0.083	0.446		

Note: CMC_net_ represents the similarity between F and F_net_. CMC_pro_ represents the similarity between F and F_pro_. ^a^ *p*-value for the main effect of comparison in a two-way mixed factorial ANOVA. ^b^ *p*-value for pairwise comparisons when interactions were detected. ^c^ *p*-value for the main effect of speed in a two-way mixed factorial ANOVA. ^d^ *p*-value for the simple effect of speed when interactions were detected. ^e^ *p*-value for the main effect of incline in a two-way mixed factorial ANOVA. ^f^ *p*-value for the simple effect of incline when interactions were detected. ^1^ Significantly different from 13 km/h. ^2^ Significantly different from 15 km/h. ^3^ Significantly different from 17 km/h. * Significantly different from 3°.

**Table 2:** Mean and standard deviation of the mean absolute difference for the DP technique (*n* = 9) and V2 technique (*n* = 10) (BW%).

		DP Technique				V2 Technique			
		$M_{\left|F_{\mathrm{net}}-F\right|}$	$M_{\left|F_{\mathrm{pro}}-F\right|}$	*p*-Value	Pη^2^	$M_{\left|F_{\mathrm{net}}-F\right|}$	$M_{\left|F_{\mathrm{pro}}-F\right|}$	*p*-Value	Pη^2^
Speed	13 km/h	6.1 ± 1.1	8.1 ± 2.9	0.058 ^b^	0.207	2.9 ± 0.4	4.4 ± 0.8		
	15 km/h	6.9 ± 1.1	9.1 ± 2.6 ^4^	0.028 ^b^	0.268	3.1 ± 0.5	4.7 ± 0.4		
	17 km/h	8.5 ± 1.5 ^1,2,5^	10.2 ± 3.3 ^1,4^	0.166 ^b^	0.116	3.6 ± 0.6	4.8 ± 0.5	0.001 ^a^	0.633
	19 km/h	9.0 ± 1.3 ^1,2^	11.6 ± 3.6 ^1,2,3^	0.057 ^b^	0.209	4.0 ± 0.9	5.4 ± 0.8		
	21 km/h	10.8 ± 2.2 ^1,2,3^	10.9 ± 2.4 ^1^	0.992 ^b^	0.001	4.4 ± 0.9	5.3 ± 0.5		
	*p*-value	0.001 ^d^	0.001 ^d^			0.001 ^c^			
	Pη^2^	0.856	0.857			0.588			
Inclines	3°	6.2 ± 0.9	8.7 ± 3.0			2.9 ± 0.5	4.5 ± 0.8	0.001 ^b^	0.617
	4°	7.1 ± 1.1	9.6 ± 2.7	0.015 ^a^	0.315	3.4 ± 1.1	4.5 ± 0.6	0.013 ^b^	0.295
	5°	7.6 ± 1.3	10.7 ± 2.9			3.7 ± 0.7 *	4.3 ± 0.9	0.115 ^b^	0.132
	*p*-value	0.001 ^e^				0.014 ^f^	0.577 ^f^		
	Pη^2^	0.615				0.394	0.063		

Note: $M_{\left|F_{\mathrm{net}}-F\right|}$ represents the absolute difference between F and F_net_ and is calculated by $\left|F-F_{\mathrm{net}}\right|$. $M_{\left|F_{\mathrm{pro}}-F\right|}$ represents the absolute difference between F and F_pro_ and is calculated by $\left|F\;-{\;F}_{\mathrm{pro}}\right|$. ^a^ *p*-value for the main effect of comparison in a two-way mixed factorial ANOVA. ^b^ *p*-value for pairwise comparisons when interactions were detected. ^c^ *p*-value for the main effect of speed in a two-way mixed factorial ANOVA. ^d^ *p*-value for the simple effect of speed when interactions were detected. ^e^ *p*-value for the main effect of incline in a two-way mixed factorial ANOVA. ^f^ *p*-value for the simple effect of incline when interactions were detected. ^1^ Significantly different from 13 km/h. ^2^ Significantly different from 15 km/h. ^3^ Significantly different from 17 km/h. ^4^ Significantly different from 19 km/h. ^5^ Significantly different from 21 km/h. * Significantly different from 3°.

**Figure A1:**
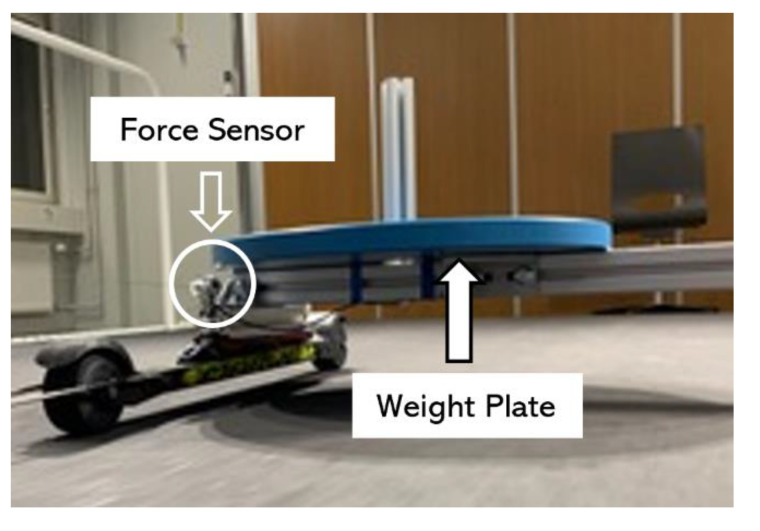
Custom-made friction measurement device.

**Figure B1:**
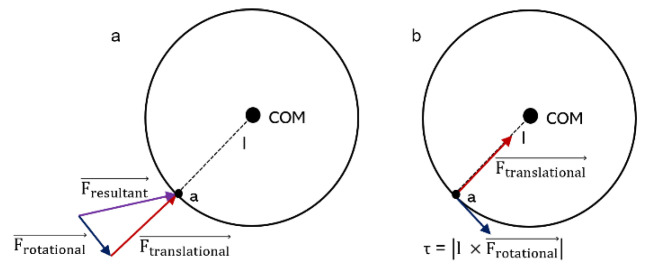
Diagram of the mechanical principle of translational force.

**Figure B2:**
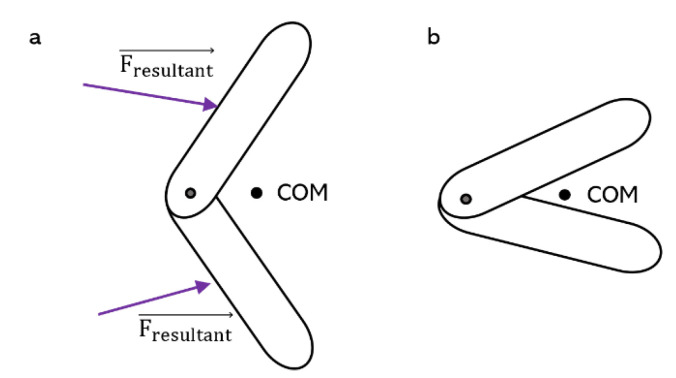
Diagram of forces acting on connected rigid bodies.

## Data Availability

The data presented in this study are available on request from the corresponding author. The data are not publicly available because the data also form part of ongoing studies.

## Appendix A. Measurement and Calculation of Resistance Friction Coefficient of Roller Ski

The resistance friction coefficient of roller ski was measured on the treadmill surface using a custom-made friction measurement device (University of Jyväskylä, Jyväskylä, Finland, Figure A1) and calculated with the LabVIEW software package (National Instruments, Austin, TX, USA) before the measurement. A commercial force sensor (Raute precision TB5, Nastola, Finland) that measures the anterior–posterior force along the roller ski (F_Y_) was contained in the friction measurement device. The friction coefficient between the treadmill surface and the roller ski was obtained by $\mu =\frac{F_{Y}}{F_{Z}}$, where F_Z_ is the vertical force that equals the weight of the weight plate placed on the roller ski.

## Appendix B. Mechanical Principle of Translational Force

The motion of a rigid body under external forces can be reduced to (i) the acceleration of the COM and to (ii) the angular acceleration of the object around its COM. These change the rate of momentum and angular momentum, respectively. Correspondingly, in a 3D space, the motion problem involves six degrees of freedom (DoF). These DoFs can be expressed with three components of a translational force and another three components of a moment. The forces and torques make a rigid body translate and rotate. It is worth noting that it is a modeler’s decision to express the six DoFs with translational forces and torques with respect to the COM. Accordingly, this is not the only, but rather a practical, option to model motion. The decomposition of an external force into translational force and torque components acting on a rigid object is illustrated in Figure B1. An external force $\vec{F_{\mathrm{resultant}}}$ acts on point a of a rigid sphere. $\vec{F_{\mathrm{resultant}}}$ is decomposed to the translational component $\vec{F_{\mathrm{translational}}}$ that acts in the direction of the line joining the COM and point a. The (displacement) vector from COM to point a is denoted by l. The rotational component $\vec{F_{\mathrm{rotational}}}$ of the force is perpendicular to vector l such that condition $\vec{F_{\mathrm{resultant}}}$ = $\vec{F_{\mathrm{rotational}}\;}$ + $\vec{F_{\mathrm{translational}}}$ holds (Figure B1a). Precisely, the same situation is expressed in terms of a translational force $\vec{F_{\mathrm{translational}}}$ and torque **τ**, which is the product of l and $\vec{F_{\mathrm{rotational}}\;}$ (Figure B1b).

As the principles of mechanics do not depend on the object—that is, Newton’s laws apply to all objects—the basic setting does not change from that of a single rigid object. However, in the case of joined bodies, the COM cannot be specified a priori, as it depends on the position of the parts in relation to each other (see Figure B2). When external forces $\vec{F_{\mathrm{resultant}}}$ act on the object (Figure B2a), the whole object translates, and each part moves with respect to each other. Because of this, the COM moves with respect to the parts. However, the motion of the object still fulfils Newton’s laws (Figure B2b). Consequently, nothing prevents one from decomposing the external forces into components of translational forces and torques with respect to the COM.

## References

[1] Smith G.A., Rusko H. (2003). Biomechanics of cross country skiing. Cross Country Skiing. Handbook of Sports Medicine.

[2] Mapelli A., Zago M., Fusini L., Galante D., Colombo A., Sforza C. (2014). Validation of a protocol for the estimation of three-dimensional body center of mass kinematics in sport. Gait Posture.

[3] Stoggl T., Holmberg H.C. (2015). Three-dimensional Force and Kinematic Interactions in V1 Skating at High Speeds. Med. Sci. Sports Exerc..

[4] Smith G., Kvamme B., Jakobsen V. Ski skating technique choice: Mechanical and physiological factors affecting performance. Proceedings of the ISBS-Conference Proceedings Archive.

[5] Hoset M., Rognstad A., Rølvåg T., Ettema G., Sandbakk Ø. (2014). Construction of an instrumented roller ski and validation of three-dimensional forces in the skating technique. Sports Eng..

[6] Göpfert C., Pohjola M.V., Linnamo V., Ohtonen O., Rapp W., Lindinger S.J. (2017). Forward acceleration of the centre of mass during ski skating calculated from force and motion capture data. Sports Eng..

[7] Ohtonen O., Lindinger S.J., Gopfert C., Rapp W., Linnamo V. (2018). Changes in biomechanics of skiing at maximal velocity caused by simulated 20-km skiing race using V2 skating technique. Scand. J. Med. Sci. Sports.

[8] Smith G.A. (1992). Biomechanical analysis of cross-country skiing techniques. Med. Sci. Sports Exerc..

[9] Holmberg H.C., Lindinger S., Stöggl T., Björklund G., Müller E. (2006). Contribution of the legs to double-poling performance in elite cross-country skiers. Med. Sci. Sports Exerc..

[10] Andersson E., Stoggl T., Pellegrini B., Sandbakk O., Ettema G., Holmberg H.C. (2014). Biomechanical analysis of the herringbone technique as employed by elite cross-country skiers. Scand. J. Med. Sci. Sports.

[11] Stoggl T., Muller E., Lindinger S. (2008). Biomechanical comparison of the double-push technique and the conventional skate skiing technique in cross-country sprint skiing. J. Sports Sci..

[12] Stoggl T.L., Holmberg H.C. (2016). Double-Poling Biomechanics of Elite Cross-country Skiers: Flat versus Uphill Terrain. Med. Sci. Sports Exerc..

[13] Sandbakk O., Holmberg H.C. (2014). A reappraisal of success factors for Olympic cross-country skiing. Int. J. Sports Physiol. Perform..

[14] Stöggl T., Stöggl J., Müller E., Erich Müller S.L., Thomas S. (2008). Competition analysis of the last decade (1996–2008) in crosscountry skiing. Science and Skiing IV.

[15] Ohtonen O., Lindinger S., Lemmettylä T., Seppälä S., Linnamo V. (2013). Validation of portable 2D force binding systems for cross-country skiing. Sports Eng..

[16] Ohtonen O., Ruotsalainen K., Mikkonen P., Heikkinen T., Hakkarainen A., Leppävuori A., Linnamo V. Online feedback system for athletes and coaches. Proceedings of the 3rd International Congress on Science and Nordic Skiing.

[17] Yu B., Gabriel D., Noble L., An K.-N. (1999). Estimate of the optimum cutoff frequency for the Butterworth low-pass digital filter. J. Appl. Biomech..

[18] Selbie W.S., Hamill J., Kepple M.T., Robertson G.E., Caldwell G.E., Hamill J., Kamen G., Whittlesey S. (2013). Three-Dimentional Kinetics. Research Methods in Biomechanics.

[19] Danielsen J., Sandbakk Ø., McGhie D., Ettema G. (2019). Mechanical energetics and dynamics of uphill double-poling on roller-skis at different incline-speed combinations. PLoS ONE.

[20] Winter D.A. (1995). Human balance and posture control during standing and walking. Gait Posture.

[21] Kadaba M., Ramakrishnan H., Wootten M., Gainey J., Gorton G., Cochran G. (1989). Repeatability of kinematic, kinetic, and electromyographic data in normal adult gait. J. Orthop. Res..

[22] Yu B., Kienbacher T., Growney E.S., Johnson M.E., An K.N. (1997). Reproducibility of the kinematics and kinetics of the lower extremity during normal stair-climbing. J. Orthop. Res..

[23] Malek M.H., Coburn J.W., Marelich W.D. (2018). Advanced Statistics for Kinesiology and Exercise Science: A Practical Guide to ANOVA and Regression Analyses.

[24] Robertson G.E., Robertson G.E., Caldwell G.E., Hamill J., Kamen G., Whittlesey S. (2013). Engergy, Work, and Power. Research Methods in Biomechanics.

[25] Schwameder H. (2008). Biomechanics research in ski jumping, 1991–2006. Sports Biomech..

